# Evaluation of the concordance of asthma remission definitions according to international guidelines in a cohort of patients treated with monoclonal antibodies for severe asthma

**DOI:** 10.3389/fphar.2026.1776527

**Published:** 2026-03-12

**Authors:** A. G. Ledda, G. Costanzo, S. Canalis, M. V. Puci, G. Sambugaro, G. Taurisano, E. Piano, M. Bullita, G. Sotgiu, D. Firinu, S. Del Giacco

**Affiliations:** 1 Department of Medical Sciences and Public Health, University of Cagliari, Cagliari, Italy; 2 Clinical Epidemiology and Medical Statistics Unit, Department of Medicine, Surgery and Pharmacy, University of Sassari, Sassari, Italy

**Keywords:** asthma, asthma remission, benralizumab, dupilumab, mepolizumab, omalizumab, severe asthma

## Abstract

**Background:**

The availability of biological therapies has helped achieve clinical remission (CR) in patients with severe asthma (SA). Scientific societies issued different definitions of remission, with the common denominator of the suspension of systemic corticosteroids for at least 1 year. Additional criteria are associated with symptoms, stability of pulmonary function, disease exacerbations, ACT ≥20 or ≥23, and ACQ ≤1.5. We evaluated the agreement among criteria adopted in Italy, Spain, Germany, Japan.

**Method:**

An observational prospective, single-center study was carried out: 65 patients with SA treated for 12 or 24 months with omalizumab, mepolizumab, benralizumab or dupilumab were recruited. Data on exacerbations, need of systemic corticosteroids, asthma symptoms, ACT and ACQ, pulmonary function at baseline and 12 and 24 months were collected. Differences of qualitative variables were assessed with the Chi-squared or Fisher’s exact test. Cohen’s Kappa coefficients were calculated to assess agreement between definitions of remission. The statistical significance threshold was set at *P* < 0.05. Data analysis was carried out using STATA version 17.

**Results:**

28 (43.1%) patients at 12 months and 27 (47.4%) at 24 months did not meet any criteria for CR, whereas 34 (52.3%) at 12 months and 28 (49.1%) at 24 months met at least one definition of complete CR. The agreement among the selected guidelines was substantial, with an overall Fleiss K of 0.7971 (P < 0.0001). Chronic rhinosinusitis with nasal polyps was more prevalent in patients who achieved CR, whereas gastro-esophageal reflux disease was more prevalent in patients who did not.

**Conclusion:**

Consistently with other studies only 52.3% at 12 months and 49.1% at 24 months achieved complete CR. The agreement of CR is substantial. The main limitations of the study are its monocentric nature and the poor sample size, whereas its main strengths were the evaluation both at 12 and 24 months and the adoption of four different definitions. Nonetheless, a worldwide definition could enhance the standardization of CR.

## Introduction

Asthma is a chronic inflammatory disease of the airways that affects over 330 million people worldwide and is a major cause of morbidity and mortality (WHO, 2025; Mims, 2015). For years, the cornerstone of asthma treatment has been the use of oral and inhaled corticosteroids (OCS and ICS). Although highly effective in relieving asthma symptoms, their nonspecific anti-inflammatory mechanism does not significantly alter the long-term natural history of the disease (Menzies-Gow et al., 2020). To date, the main goals of asthma treatment have been to reduce exacerbations and achieve symptom control. In recent years, several monoclonal antibodies (mAbs) have been developed and used as add-on therapy for patients with severe asthma, demonstrating effectiveness in reducing exacerbations and OCS use, as well as improving lung function and quality of life (QoL). The advent of these novel therapeutic options for severe asthma has opened the possibility of setting a new goal, similar to what is pursued in other chronic inflammatory diseases such as rheumatoid arthritis and inflammatory bowel diseases: on-treatment disease remission (Menzies-Gow et al., 2020). Remission is not an entirely new concept in asthma, as physiological remission may occur in some cases of childhood asthma as patients reach adulthood.

Since identifying or developing targeted treatments that can achieve disease remission represents a step towards a potential cure, defining this new goal is very relevant (Thomas et al., 2022).

In several countries, scientific societies have developed consensus criteria to define asthma remission and strategies to achieve it. Some of these consensus statements have been incorporated into national guidelines; however, there is currently no international agreed-upon definition. Germany was the first country to include a definition of remission in its national guidelines as an overarching goal of asthma management, defining clinical remission as the fulfilment of four criteria for at least 12 months: absence of exacerbations; no use of OCS for asthma control; no asthma-related symptoms; and stable lung function (Lommatzsch et al., 2023).

The Spanish guidelines, published in May 2023, propose two types of remission: clinical remission, defined by the absence of asthma symptoms, no need for OCS, absence of exacerbations, and optimised and/or stable lung function for at least 12 months; and complete remission, which requires clinical remission plus the absence of bronchial hyperresponsiveness and airway inflammation (Plaza Moral et al., 2023). In July 2023, the Japanese guidelines also defined clinical remission using the following criteria: an asthma control test (ACT) score ≥23 points, no exacerbations, and no use of OCS for at least 12 months (lung function was not included) (Niimi et al., 2023). In August 2023, following a Delphi consensus process, the Severe Asthma Network Italy (SANI) proposed two definitions of asthma remission: partial remission, defined as no OCS use for asthma treatment and fulfilment of at least two out of three criteria (no exacerbations, stable lung function and no asthma symptoms), combined with ACT score ≥20 and an Asthma Control Questionnaire (ACQ) < 1.5, and complete clinical remission when all the criteria are met (Canonica et al., 2023) (Table 1).

**TABLE 1:** Resume of the criteria necessary to define asthma remission in national guidelines of Germany, Spain, Japan, Italy.

Criteria	Germany	Spain	Japan	Italy
No Exacerbations	✓	✓	✓	✓
No Need for OCS	✓	✓	✓	✓
No Asthma Symptoms	✓	✓	Not required	✓
Stable Lung Function	✓	✓	Not required	✓
ACT ≥20	Not required	Not required	Not required	✓
ACT ≥23	Not required	Not required	✓	Not required
ACQ <1.5	Not required	Not required	Not required	✓

OCS, Oral CorticoSteroids; ACT, asthma control test; ACQ, asthma control questionnaire.

Biologic drugs show disease-modifying activity owing to their anti-inflammatory mechanism (Busse et al., 2022). However, comorbidities in asthmatic patients must be carefully taken into consideration. They are linked to worse outcomes (*i.e.,* exacerbations, poor disease control, and reduced QoL) and a management based on a personalised approach which considers disease phenotype and environmental factors is crucial (Scelo et al., 2024). Chronic Rhinosinusitis with nasal polyposis (CRSwNP) (Laidlaw et al., 2021) and food allergy (Price et al., 2021) are often linked to asthma. In patients with severe asthma, prevalence of gastroesophageal reflux disease (GERD) ranges from 46% to 63% (Rogliani et al., 2020), (Pavord et al., 2023). Bronchiectasis is associated with increased rates of exacerbations and hospitalisations (Lan et al., 2021). Severe asthma can be concomitantly diagnosed with obstructive sleep apnoea syndrome (OSAS) and oxyhaemoglobin desaturation (Chaouki and Djebbar, 2018). Eosinophilic granulomatosis with polyangiitis (EGPA) is associated with asthma and ENT disorders occur in over 90% of EGPA (Koike et al., 2023). Asthmatic patients using high doses of corticosteroids may develop serious ocular manifestations (e.g., cataract and glaucoma) (Williamson et al., 1969). Hypertension is the most common cardiovascular comorbidity; additionally, asthma-related systemic inflammation may impact cardiovascular function (Cazzola et al., 2022). Finally, individuals with asthma have an increased risk of developing depression and anxiety (Lehrer et al., 2002), (Del Giacco et al., 2016).

### Aim of the study

The aim of the study is to assess prevalence of asthma remission as defined by German, Spanish, Japanese, and Italian guidelines in a cohort of patients treated with mAbs for at least 12 months.

## Materials and methods

In our prospective observational, single-centre, real-life study, patients with severe asthma diagnosed according to ERS/ATS guidelines (Stanojevic et al., 2022) and treated with a mAb (i.e., omalizumab, mepolizumab, benralizumab, or dupilumab) for at least 12 months were enrolled.

Inclusion criteria

- Diagnosis of Severe Asthma
- Treatment with mAb for Severe Asthma at least 12 months before the time of analysis
- Age >18 years old
- Stable treatment with moderate or high dose ICS + LABA with or without long acting muscaric antagonist (LAMA) during the time of analysis

Exclusion criteria:

- Diagnosis of chronic obstructive pulmonary disease (COPD) or asthma COPD overlap (ACO)

At the time of analysis only a subset of the cohort had reached a treatment duration with mAbs longer or equal to 24 months; consequently, it was not possible to collect the complete set of data required for long-term assessment in these cases and so only 57/65 (87.7%) patients had data collected also at 24 months. This temporal heterogeneity in treatment exposure reflects the gradual implementation of these therapies in clinical practice and represents a methodological limitation typical of real-life evidence studies. At baseline the following data were collected: age, sex, smoking habit, lung function (FEV1 at baseline, at 12 and at 24 months), additional therapy (LAMA), and comorbidities. Patients were divided into groups according to their ICS daily dose (Authors Anonymous, 2003): a) High dose: fluticasone propionate (FP) > 400 mcg/day or budesonide (BUD) > 800 mcg/day or beclometasone (BDP) b) Moderate dose: 200–400 mcg/day FP or 400–800 mcg/day BUD or BDP. Another stratification was based on serum eosinophils (EOS) count at baseline: low (<300 cells/mcl), medium (≥300 cells/mcl but <700 cells/mcl), and high (>700 cells/mcl). Remission criteria for each definition were collected at 12 months: ACT score, ACQ score, lung function (assessed according to the latest ERS/ATS guidelines (Stanojevic et al., 2022)), asthma exacerbations, need for OCS, and asthma symptoms. Stable lung function was defined as as having a reduction of FEV1 at follow-up equal or lower than minimal clinical important difference (MCID) for FEV1 (15%) (Bonini et al., 2020). The study was conducted in accordance with the Declaration of Helsinki, obtaining written consent from all participants (SANI study approved on 23–072018; #15).

### Statistical analysis

Data were summarised with mean and standard deviation (SD) or median and interquartile range (IQR) for quantitative variables and with absolute and relative (percentage) frequencies for qualitative variables. Shapiro–Wilk test was used to assess the normality of the data distribution. Differences in qualitative variables between groups were assessed by Chi-squared or Fisher exact test. Cohen’s Kappa coefficients were calculated to assess agreement between 12 months and 24 months definitions of remission. The statistical significance threshold was set at P < 0.05, and data analysis was carried out using STATA version 17.

## Results

65 patients were enrolled, with 46 (70.8%) females. The median (IQR) age was 55 (48–62) years. 49 patients were never smokers (75.4%), whereas 16 (24.6%) were former smokers. The (Table 2). A follow-up visit at 24 months was performed only for 57 (87.7%). The median disease duration before enrollment was 15 years (IQR 10–25).

**TABLE 2:** Demography of the population enrolled in the study at baseline.

Demography	
Patients enrolled (n)	65
Age, median (IQR)	55 (48–62)
Sex F (n, %)	46 (70.8)
Never smokers (n, %)	49 (75.4)
Current smokers (n, %)	0 (0)
Former smokers (n, %)	16 (24.6)
Disease duration, median (IQR)	15 (10–25)

SD, standard deviation; IQR, interquartile range.

In our cohort of 65 patients, 15 (23.08%) patients were treated with omalizumab, 15 (23.08%) with mepolizumab, 19 (29.23%) with benralizumab and 16 (24.62%) with dupilumab. They all had a 12-month follow-up. 57 patients on 65 had also a 24 follow up, of whom 14 (24.56%) patients were on omalizumab, 12 (21.05%) were on mepolizumab, 18 (31.57%) on benralizumab and 13 (22.8%) were on dupilumab (see Supplementary Material Table S1 in Supplementary Material).

The time of disease duration prior to initiation of mAb was comparable between groups of patients who achieved or not achieved each definition of asthma remission both at 12 months (p = 0.47) that at 24 months (p = 0.81).

Considering lung function, at baseline the median value of FEV1% in our cohort was or 85 (IQR 57–102), while at 12 months the median FEV1% was 93 (IQR 74–106) and at 24 months 95 (IQR 81.3–112).

**FIGURE 1:**
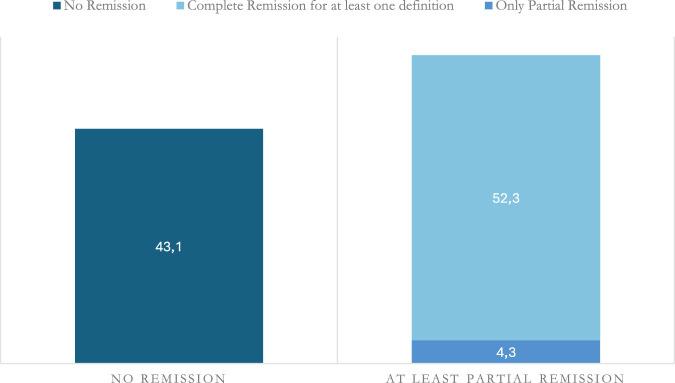
% of patients who achieved asthma remission at 12 months.

**FIGURE 2:**
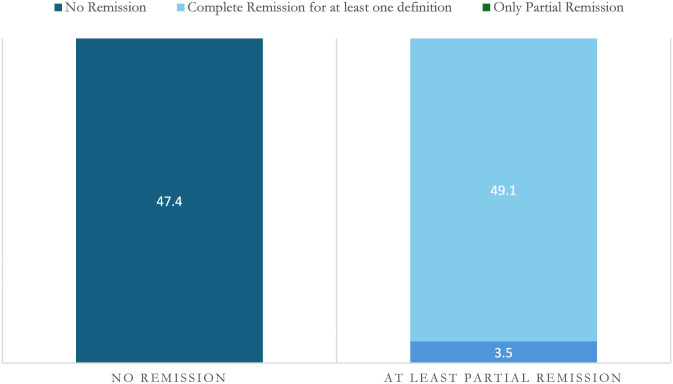
% of patients who achieved asthma remission at 24 months.

At 12 months, 28 (43.1%) did not meet the criteria for any of the definitions of asthma remission; 37 (56.9%) met the criteria for the SANI partial remission; 34 (52.3%) for complete clinical remission for at least one of the definitions considered, and 23 (35.4%) for all definitions (Figure 1). At 24 months, 27 (47.4%) did not meet the criteria for any of the definitions, whereas 30 (52.6%) for at least the SANI partial remission, 28 (49.1%) for complete clinical remission at least for one of the definitions, and 22 (38.6%) for all definitions (Figure 2).

In detail, SANI complete remission criteria were met at 12 months in 27/65 patients (41.5%), at 24 months in 26/57 (45.6%). Spanish clinical remission criteria were met in 30/65 (46.1%) at 12 months, at 24 months in 27/57 (47.3%). For the German definition of clinical remission, at 12 months criteria were met in 30/65 (46.1%), whereas at 24 months in 27/57 (47.3%). Japanese clinical remission criteria were met in 26/65 (40%) at 12 months and in 25/57 (43.8%) at 24 months (Table 3).

**TABLE 3:** n (%) of patients that achieved asthma remission at 12 and 24 months for each definition.

Definition	12 months (n=65)	24 months (n=57)
Germany	30 (46.1%)	27 (47.3%)
Spain	30 (46.1%)	27 (47.3%)
Japan	26 (40%)	25 (43.8%)
Italy Partial	37 (56.9%)	30 (52.6%)
Italy Complete	27 (41.5%)	26 (45.6%)

Analysing the agreement between criteria, we have found for the outcome “no remission” Fleiss K = 0.8522, for the outcome “remission at 12 months” Fleiss K = 0.5939, for the outcome “clinical remission at 24 months” Fleiss K 0.6926, for the outcome “remission at 12 and 24 months” Fleiss K = 0.8501, and finally for the overall agreement Fleiss K 0.7971 (Table 4).

**TABLE 4:** Concordance between the definitions considered based on the results of our cohort.

Outcome	Fleiss K	P-value
No remission	0.8522	<0.0001
Remission at 12 months	0.5939	<0.0001
Remission at 24 months	0.6926	<0.0001
Remission at 12 and 24 months	0.8501	<0.0001
Overall	0.7971	<0.0001

“No OCS” was the most frequently met criteria (69.2% at 12 and 24 months) followed by stable lung function (67.7%), whereas the less frequently met was ACT ≥23 (never achieved in 47.7%), followed by no symptoms (46.2%) (see Supplementary Material Table S2, Supplementary Materials).

Regarding comorbidities, at 12 months, CRSwNP was significantly more frequent in the group of patients that meet the criteria for at least one definition of clinical remission compared to the group who did not undergo clinical remission (89.2% VS. 60.7%; *p =* 0.007), whereas GERD was significantly more frequent in the group who did not meet the criteria for any of the definitions of asthma remission compared with the group who reached at least one definition (89.3% VS. 51.4%; *p =* 0.001) (See Supplementary Material Table S3 in Supplementary Material). At 24 months from the baseline GERD was still significantly more frequent in the group of patients who did not reach remission (85.2% VS. 50%; *p =* 0.005) (See Supplementary Material Table S4 in Supplementary Material).

No statistically significant differences were observed between the groups considering the baseline serum EOS counts, but there was a greater number of patients with medium EOS in the group of patients who reached a clinical remission (See Supplementary Material Table S5 in Supplementary Material). Considering additional therapy, in our cohort 47 out of 65 patients were treated also with LAMA for the entire duration of the study, while 18 did not take LAMA during the study period. We did not find any statistically significant difference in patients treated with or without this kind of therapy, both at 12 months (p = 0.12), than at 24 months (p = 0.16 considering 42 patients treated with LAMA vs. 15 patients without on 57 patients total).

At 12 months, 21 patients took a maintenance medium ICS dose and 44 a maintenance high dose. In the group with a medium ICS dose, a higher percentage reached asthma remission compared with the high ICS dose group, both at 12 months (76.2% VS. 47.7%; p = 0.03) and at 24 months (79% VS. 39.5%; *p =* 0.005) (See Supplementary Material Table S6 in Supplementary Material). 11 patients underwent a switch of mAb: 5 (45.5%) met the criteria for at least one definition of asthma remission at 12 months, 22.2% at 24 months (See Supplementary Material Table S7 in Supplementary Material). We found no significant statistical difference between patient who achieved or not achieved any definition of remission at 12 (p = 0.47) or 24 months (p = 0.81) months the years of disease duration pre-enrolment. We finally performed a subanalysis evaluating remission outcomes by biologic agent. We found that after 12 months of treatment 4/15 patients treated with omalizumab (26.7%), 4/15 treated with mepolizumab (26.7%), 4/19 treated with benralizumab (21.1%) and 11/16 treated with dupilumab (68.8%) achieved clinical remission for every definition (p = 0.03). After 24 months, 5/14 patients treated with omalizumab (35.7%), 4/12 treated with mepolizumab (33.3%), 5/18 treated with benralizumab (27.8%) and 8/13 treated with dupilumab (61.5%) achieved clinical remission for every definition considered (p = 0.02).

## Discussion

Our study showed that 56.9% and 52.6% of patients, at 12 and 24 months respectively, at least met the SANI criteria for partial asthma remission, with 52.3% and 49.1% at least one definition of complete clinical remission, whereas only 35.4% and 28.6% at 24 all definitions of clinical remission. Ultimately, 43.1% and 43.4% could not be classified as in remission. Those findings align with those reported in literature. An observational study carried out in Australian patients treated with omalizumab for severe asthma and published in 2023, showed that of 175 patients enrolled, 22.8% achieved clinical remission at 12 months, while 19.1% achieved remission and stabilisation or optimisation of lung function (Thomas et al., 2024). Another study conducted on 302 Italian patients treated with Omalizumab for 1 year showed a clinical remission rate of 21.8% (Sposato et al., 2023).

A post-hoc analysis of the REDES trial evaluated asthma remission in patients treated with Mepolizumab, using two definitions of “on-treatment” clinical remission, and showed that out of 144 patients evaluated, 30% met the first definition, while out of 260 patients, 37% met the second definition (Pavord et al., 2023). Regarding benralizumab, a post hoc analysis evaluated remission rates using data from the SIROCCO, CALIMA, and ZONDA studies showed that at 12 months after the start of therapy, 48.5% (284/586) and 14.5% (85/586) achieved at least 3 and all 4 criteria, respectively (Menzies-Gow et al., 2022a). Another Italian real-life study performed on 164 comorbid patients with severe asthma plus CRSwNP found a remission rate of 42.1% at 24 months after initiation of benralizumab therapy (Pelaia et al., 2024).

A post hoc analysis of the QUEST and TRAVERSE trials evaluated clinical remission on data obtained from these clinical trials. In total, 37.2% of QUEST patients treated with dupilumab and 42.8% of QUEST + TRAVERSE patients treated with dupilumab achieved clinical remission (Pavord et al., 2025). A Dutch study in 2024 found in a cohort of 136 patients treated with Dupilumab a clinical remission rate of 21.3%. (Pavord et al., 2025), (Bult et al., 2024), (Chipps et al., 2025). Finally another study performed in the United States involving 611 patients treated with omalizumab, mepolizumab, benralizumab, reslizumab, tezepelumab, or dupilumab showed that only 46% of participants satisfied the criteria for asthma remission (as determined by clinical opinion and, within the past year: no asthma exacerbations, no requirement for oral corticosteroids to manage asthma, and an ACT score exceeding 20 in at least half of the assessments conducted) (Chipps et al., 2025).

The results of these studies, even if they considered two slightly different definitions of asthma remission and enrolled a greater cohort of patients, are in agreement with ours about the fact that only a selected number of patients achieved asthma remission.

The definition achieved more often was SANI partial remission (56.9% and 53.3% at 12 and 24 months, respectively), because of its somehow less restrictive definition, followed by the Spanish and German definitions of clinical remission (46.1% and 47.3% at 12 and 24 months, respectively), and the SANI definition of complete remission (41.5% and 45.6% at 12 and 24 months, respectively), which is more restrictive due to inclusion of ACT and ACQ scores. Japanese guidelines were the least achieved (40% and 43.8% at 12 and 24 months, respectively).

Analysing the agreement between criteria, we have found an almost perfect agreement for the outcome “remission at 12 and 24 months” (Fleiss K: 0.8501) and for the outcome “no remission” (Fleiss K: 0.8522); a substantial agreement, if the overall agreement (Fleiss K 0.7971) or the outcome “clinical remission at 24 months” (Fleiss K 0.6926) were considered; a moderate agreement considering the outcome “clinical remission at 12 months” (Fleiss K 0.5939). Considering these results, we could say that patients who do not achieve remission according to one of the guidelines we evaluated, in general also fail to attain remission according to the others. This indicates that the guidelines share analogous cornerstones, particularly the “no OCS” criterion, which implies that a patient failing to achieve this condition cannot be classified as in remission according to any of the evaluated guidelines.

In fact, considering the guidelines criteria, we have found that the most frequently fulfilled are “no need for OCS” (69.2%), followed by “stable lung function” (67.7%).

The criteria less commonly met are “ACT ≥23” (47.7%) and “no symptoms in the last 12 months” (46.2%). In particular “ACT ≥23” had an important impact on remission compared with “no symptoms”. In fact, while 38.46% of patients fulfilled “no symptoms” both at 12 than at 24 months, only 29.23% fulfilled the criteria “ACT ≥23” at the same time points, showing that the japanese definition appears to be the most strict.

Regarding comorbidities, we have found that CRSwNP was significantly more frequent in patients that met the criteria for at least one definition of clinical remission at 12 months (89.2% vs. 60.7% p = 0.007), but not at 24 months. GERD was significantly more frequent in the group of patients who do not fulfil any definition of clinical remission both at 12 months (89.3% vs. 51.4% p = 0.001) and at 24 months (85.2% vs. 50% p = 0.005). It is known that CRSwNP is tightly associated with SA; in fact, 51%–67% of patients with SA have CRSwNP as a comorbidity. Patients with both SA and CRSwNP, usually show an higher inflammation in the lower airways and worse asthma control. Additionally, poorer asthma control results in poorer CRSwNP control, and vice versa (Laidlaw et al., 2021). The larger number of patients in clinical remission having both SA and CRSwNP may be linked to a lower control of asthma before starting biologic treatment in a condition of comorbidity which may be mutual triggering. It’s also true that patients with both SA and CRSwNP are often affected by a T2-high inflammation that is known to have the best response to mAbs for SA treatment. Approximately 52.7% of people with asthma also suffer from GERD. Data shows that people with GERD experience poorer control of asthma, higher bronchial reactivity, reduced quality of life, and an increased frequency of exacerbations. The mechanism underlying this phenomenon remains unclear, potentially including a direct process (micro aspirations of acid reflux into the bronchi) or an indirect one (stimulation of the vague nerve) (McDonald et al., 2024). Consequently, the higher number of patients with GERD in the non-remission group can be interpreted as indicative of this poorly controlled trait.

Patients treated with medium ICS dose achieved remission more frequently compared with those treated with a higher ICS dose, mirroring the severity of their disease.

Finally, we performed a sub-analysis on switchers. Data from several countries, including Kuwait, Bulgaria, Canada, Denmark, South Korea, Italy, and Greece, show that many patients discontinue or switch their biologic therapy within the first year of treatment (Menzies-Gow et al., 2022b). In our cohort 11 out of 65 patients had a mAb switch due to various factors, including lack of response and adverse effects. Considering these 11 patients, after 12 months from switch, 45.5% met at least one definition of complete clinical remission. At 24 months, the rate was only 22.2%, calculated from a smaller cohort of 9 patients. Consequently, following the switch, the remission rate appears to be comparable to that of the naive population at 12 months, then decrease at 24 months. This underlines that despite the current mAbs being all effective against type 2 inflammation, a careful individual phenotyping is needed before starting the mAb with therapeutic switch being crucial tool for addressing non- or low-responder patients, even if in a certain percentage of patients this strategy is not sufficient to achieve disease remission. However, we should interpret these results cautiously, given the limited patient sample size. Regarding the evaluation of remission outcomes by mAb, we found that patients treated with dupilumab achieved remission considering all the definitions more frequently both at 12 months and at 24 months, compared with patients treated with other mAbs. Given the small sample size, these findings should be interpreted with caution.

Finally, neither treatment with LAMA nor disease’s duration prior to mAb therapy affected asthma remission rate in our cohort of patients. This real-world study presents some limitations, primarily related to its observational design and the relatively small sample size. However, compared to studies addressing a similar topic, the current research presents several strengths, including the evaluation of patients at both 12 and 24 months, the assessment on four monoclonal antibodies, and the inclusion of four international definitions of asthma remission.

## Conclusion

A significant proportion of patients treated with mAbs for SA do not achieve clinical remission after 12 and 24 months of therapy. They showed a reduction of the average dose of OCS and an improvement or stability of the lung function. The main guidelines show agreement, especially for the absence of clinical remission. Finally, management of comorbidities and treatable traits are key to achieve asthma control, and a therapeutic switch can be effective in case of insufficient or partial response to a mAb.

## Data Availability

The raw data supporting the conclusions of this article will be made available by the authors, without undue reservation.
