# Supplementary material for: Evaluation of the concordance of asthma remission definitions according to international guidelines in a cohort of patients treated with monoclonal antibodies for severe asthma
Source: Front Pharmacol. 2026 Mar 12;17:1776527. doi: 10.3389/fphar.2026.1776527 (PMC13018167; doi:10.3389/fphar.2026.1776527)

**Supplementary materials**

Table 1S. n (%) of patients treated with each mAb during the study and at each timepoint

**
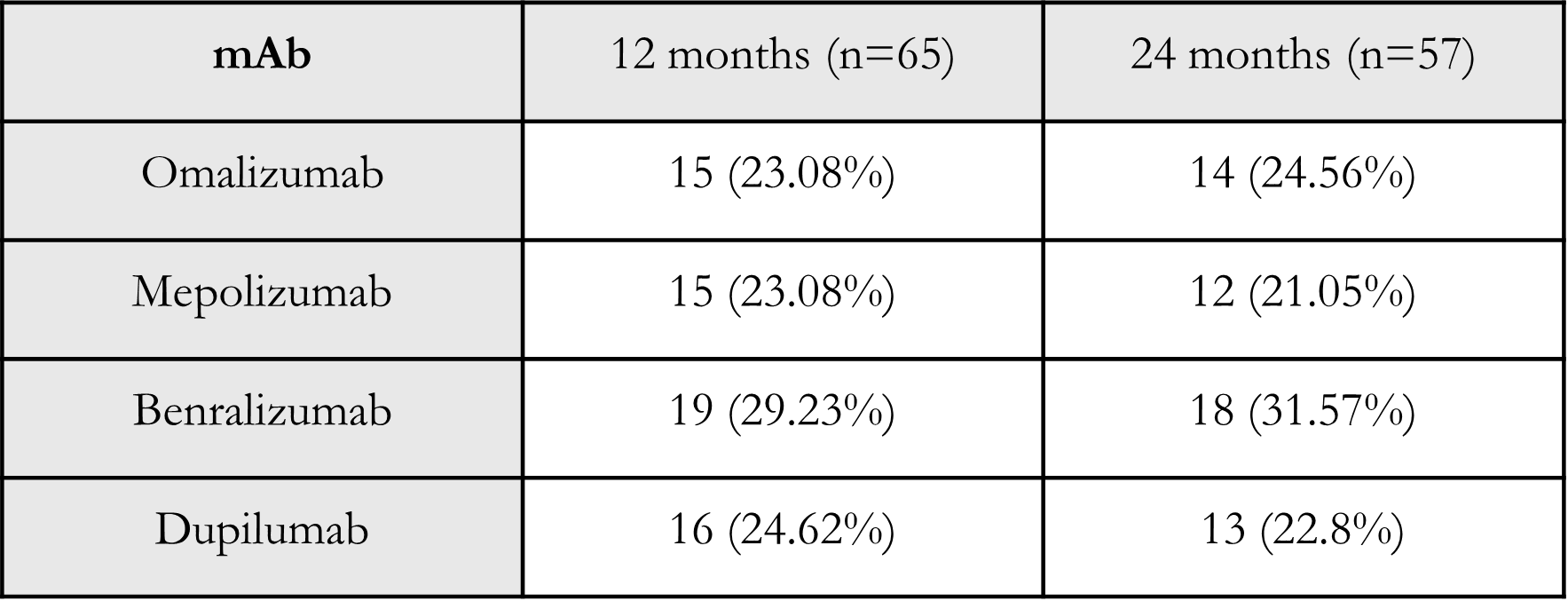
**mAb: monoclonal Antibody

Table 2S: Frequency of achievement for each criteria of the definition considered


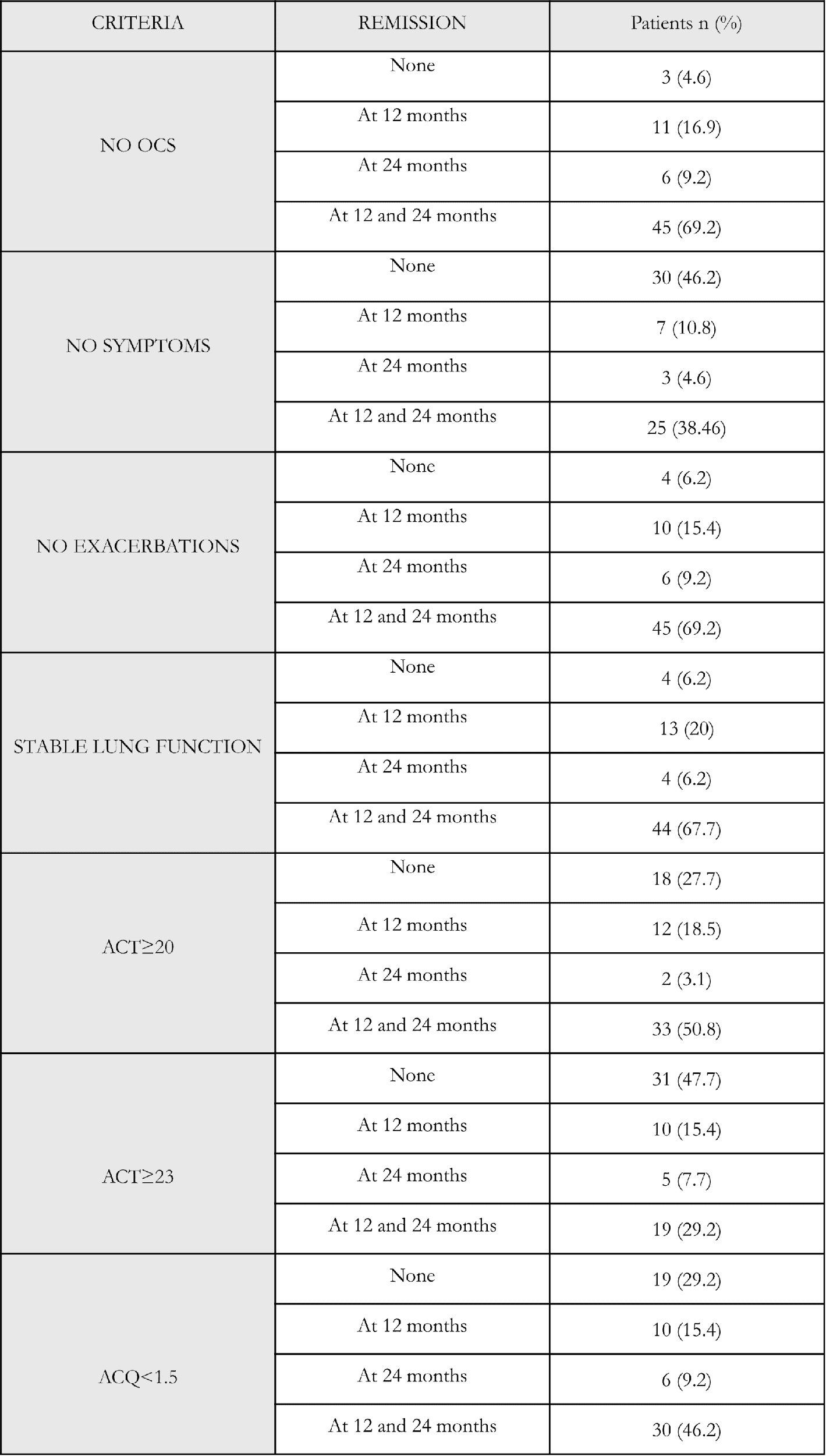


OCS: Oral Corticosteroids; ACT: Asthma Control Test; ACQ: Asthma Control Questionnaire

Table 3S: Frequency of the main asthma comorbidities in our cohort of patients at 12 months


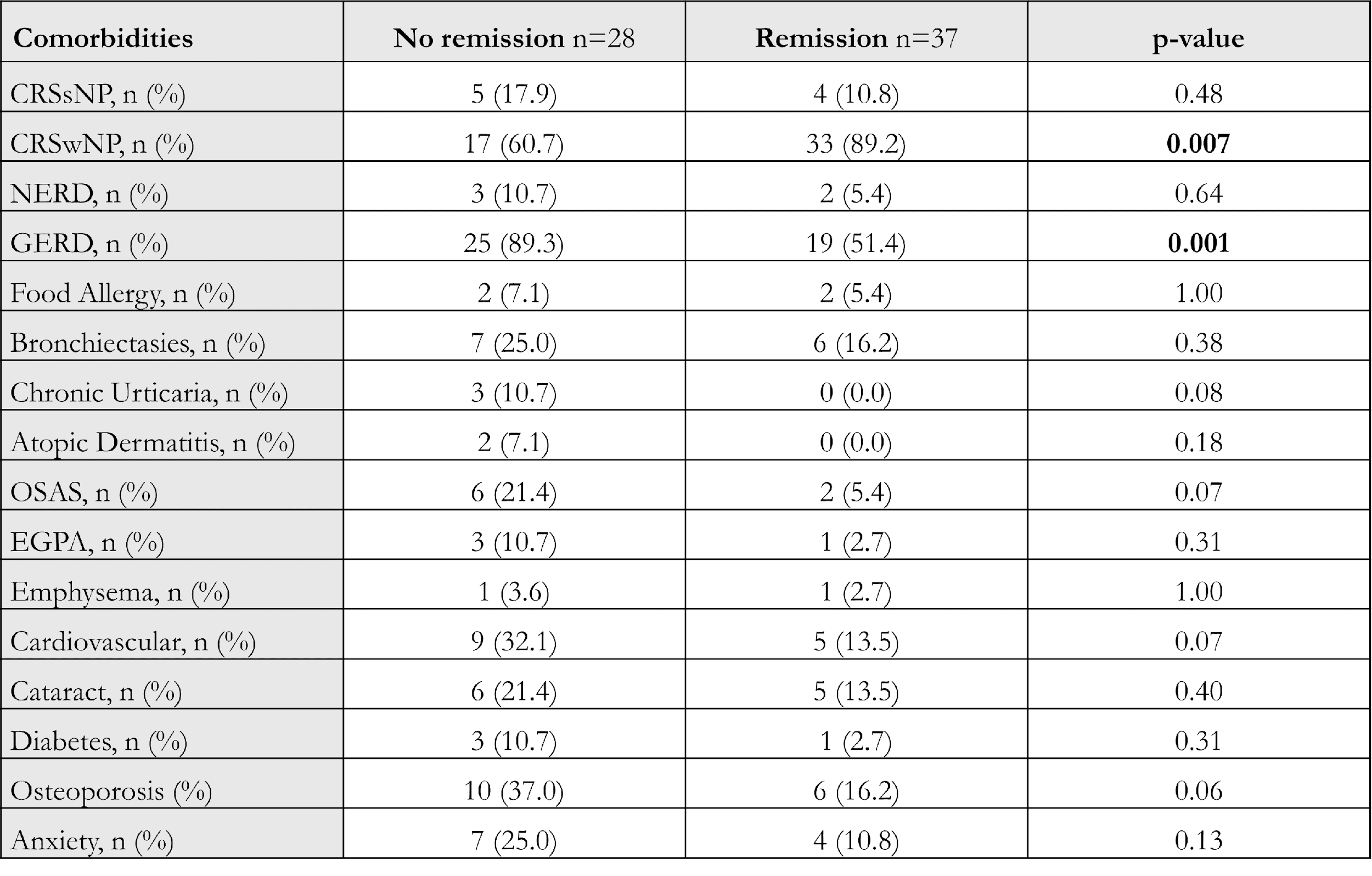


CRSsNP: Chronic Rhinosinusitis without Nasal Polyps; CRSwNP: Chronic Rhinosinusitis with Nasal Polyps; NERD: NSAID-Exacerbated Respiratory Disease: GERD: Gastroesophageal Reflux Disease; OSAS: Obstructive Sleep Apnea Syndrome; EGPA: Eosinophilic Granulomatosis with polyangiitis

Table 4S: Frequency of the main asthma comorbidities in our cohort of patients at 24 months


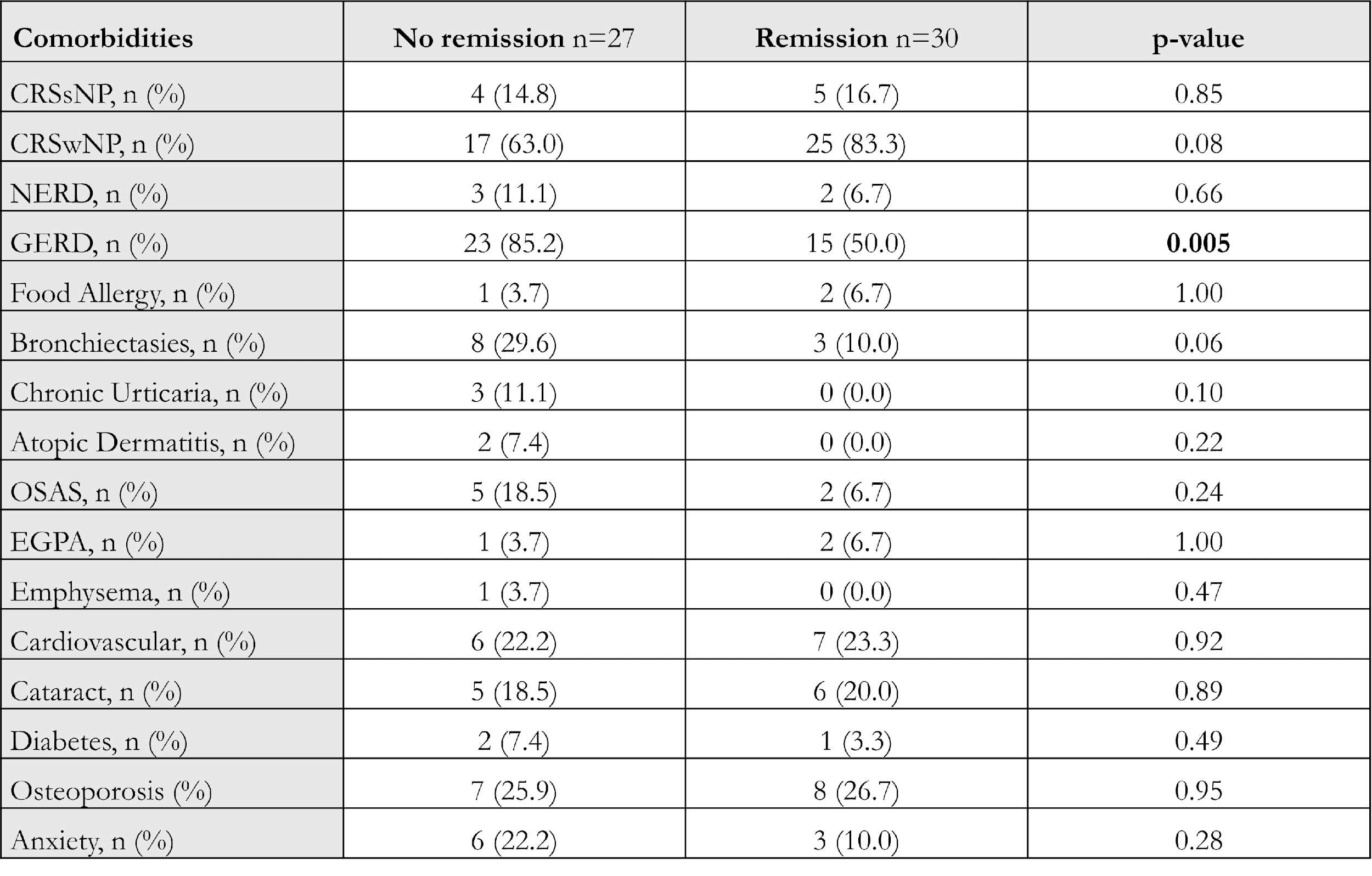


CRSsNP: Chronic Rhinosinusitis without Nasal Polyps; CRSwNP: Chronic Rhinosinusitis with Nasal Polyps; NERD: NSAID-Exacerbated Respiratory Disease: GERD: Gastroesophageal Reflux Disease; OSAS: Obstructive Sleep Apnea Syndrome; EGPA: Eosinophilic Granulomatosis with polyangiitis

Table 5S: Baseline blood eosinophils count in patients who achieved remission at 12 and 24 months


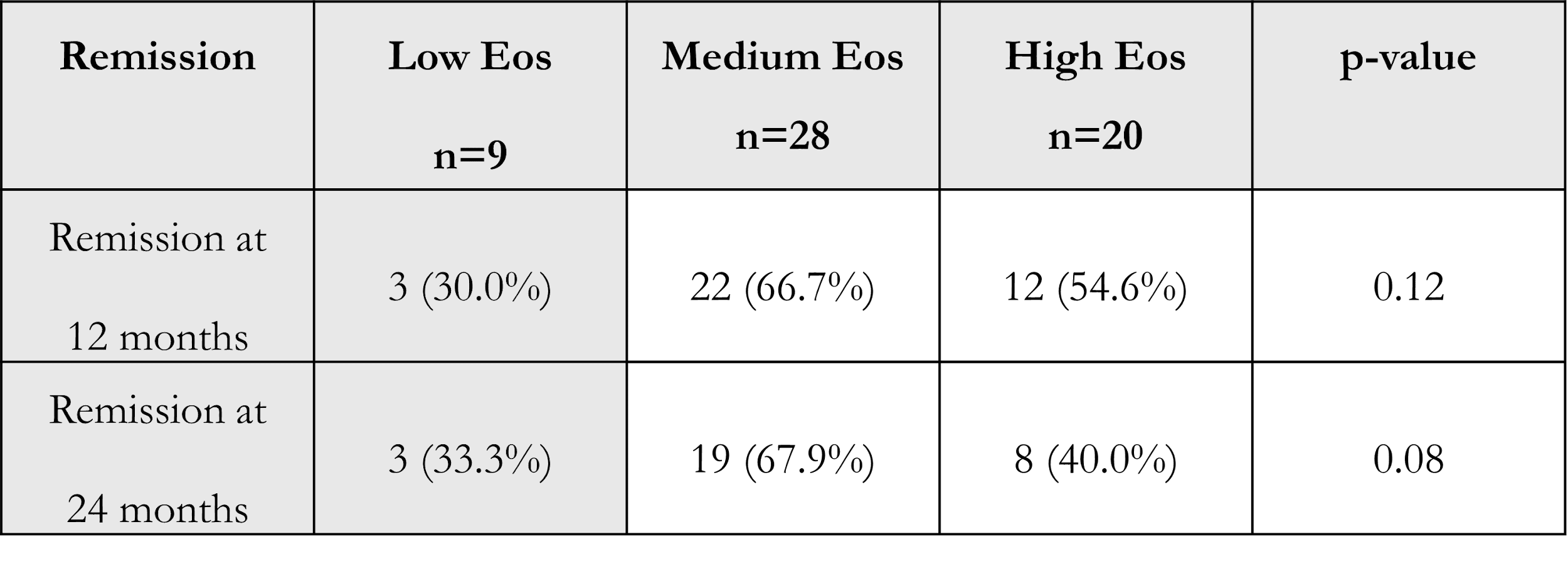


Eos: Baseline blood eosinophils count

Table 6S: Remission at 12 and 24 months based on ICS dose


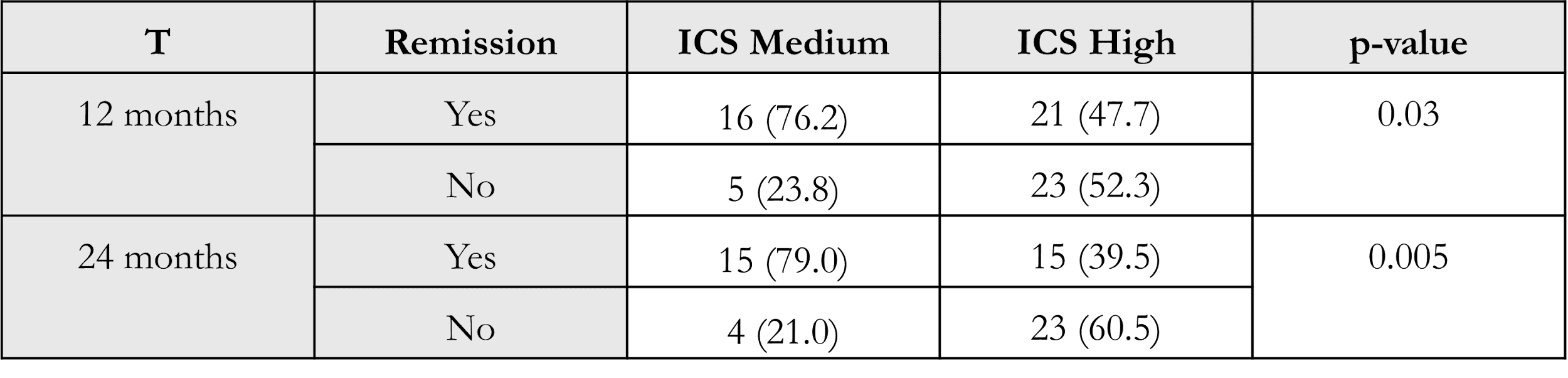


ICS: Inhaled Corticosteroids

Table 7S: Percentage of patients who or who not achieved asthma remission at 12 and 24 months after switching mAb


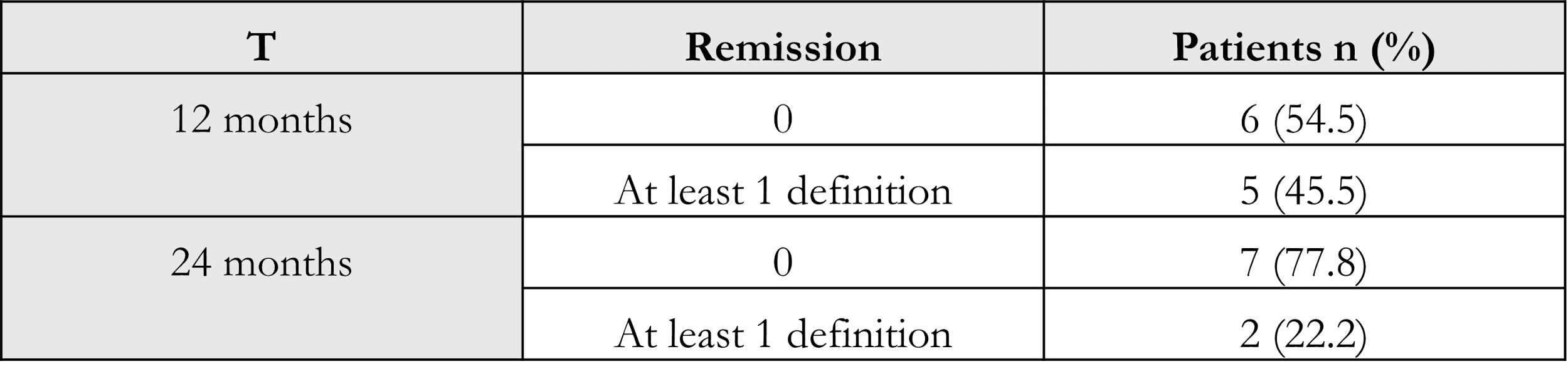

Supplement: Supplementary file 1 [file Table1.docx]
